# Concomitant use of renin-angiotensin-aldosterone system inhibitors prevent trastuzumab-induced cardiotoxicity in HER2+ breast cancer patients: an institutional retrospective study

**DOI:** 10.1186/s40959-019-0043-8

**Published:** 2019-07-08

**Authors:** Melissa Y. Y. Moey, Darla K. Liles, Blase A. Carabello

**Affiliations:** 1 Department of Internal Medicine, Vidant Medical Center/East Carolina University, 2100 Stantonsburg Road, Greenville, North Carolina 27834 USA; 2 Department of Hematology and Oncology, Vidant Medical Center/East Carolina University, 2100 Stantonsburg Road, Greenville, North Carolina 27834 USA; 3 Department of Cardiology, Vidant Medical Center/East Carolina University, 2100 Stantonsburg Road, Greenville, North Carolina 27834 USA

**Keywords:** Cardiotoxicity, Herceptin, Trastuzumab, Angiotensin converting enzyme inhibitors, Beta-blockers, Angiotensin receptor blockers

## Abstract

**Background:**

Cardiotoxicity is an adverse effect of trastuzumab (TRA) in the treatment of human epidermal growth factor 2 positive (HER2+) breast cancer. Current literature on the cardioprotective effects of agents targeted against the renin-angiotensin-aldosterone system (RAAS) and beta-blockers (BB) in TRA-treated HER2+ breast cancer patients is conflicting. We hypothesized that concurrent use of RAAS inhibitors would prevent TRA-induced cardiotoxicity (TIC).

**Methods and materials:**

Surveillance ejection fraction (EF) at 3-month intervals up to 36 months obtained from echocardiogram or multigated acquisition (MUGA) scans were retrospectively compared to baseline EF in TRA-treated HER2+ breast cancer patients between 2011 to 2016 at a tertiary cancer center. TIC was defined as a decrease of EF by more than 15 EF percentage points from baseline on surveillance imaging. Cardiac medications and comorbidities were compared between patients with reduced EF secondary to TIC (rEF) and patients who did not experience TIC (pEF). A published clinical risk score (CRS) was applied to the patient population with calculated sensitivity analyses to determine if the CRS could predict TIC.

**Results:**

Of 127 patients with TRA-treated HER2+ breast cancer, 11% developed cardiotoxicity resulting in discontinuation of TRA. Cardiotoxicity with reduced EF was seen as early as 3 months and at subsequent 3-month follow up intervals up to the 15-month follow-up. Co-existing arrhythmia, coronary artery disease (CAD), hypertension (HTN) and diabetes mellitus (DM) tended to infer an increased risk for cardiotoxicity. Patients with pEF were found to be concurrently on a RAAS inhibitor more than the rEF group (OR of 0.24, 95% CI 0.05–1.11, p 0.06). The CRS high-risk cut-off had a sensitivity of 0.17 (95% CI 0.03–0.49), specificity of 0.89 (95% CI 0.82–0.94), positive predictive value of 0.14 (95% CI 0.03–0.44) and negative predictive value of 0.91 (95% CI 0.84–0.95).

**Conclusion:**

Our data suggest that the concurrent use of a RAAS inhibitors during TRA treatment may provide a protective effect against TIC and warrants further investigation. The low sensitivity and positive predictive value demonstrated that the CRS has minimal utility as a screening tool for prediction of patients at high risk for TIC. Therefore, closer surveillance of patients receiving TRA is warranted for early detection of TIC.

## Introduction

Since the discovery of the human epidermal growth factor receptor 2 (HER2)/*neu* oncogene by Weinberg and colleagues in 1984 [1], HER2 has been the focus of targeted pharmacotherapy in HER2 positive (HER2+) early and metastatic breast cancer. Overexpression of HER2 is found in at least 20–25% of all breast cancer [2] and is responsible for increased aggressiveness via several pro-oncogenic and anti-apoptotic pathways [3]. Trastuzumab (TRA) is a monoclonal IgG κ antibody against the extracellular domain of HER2 that when administered with cytotoxic chemotherapy as adjuvant therapy or in the metastatic setting significantly reduces tumour size and increases survival rates [4, 5].

The increase in overall survival in early and metastatic breast cancer patients receiving TRA adjunctive therapy however was not without consequence. A major adverse effect observed with TRA therapy was the development of symptomatic heart failure in up to 7% of patients and asymptomatic decrease in ejection fraction (EF) between 0.7 to 19% of patients [6–10]. The prospective 5-year survival rate of > 70% with breast cancer is consequently reduced to < 50% as some patients continue to experience persistent cardiac dysfunction [11, 12]. Given the known adverse effect of TRA on cardiac function, the FDA recommends surveillance cardiac imaging at an interval of 3 months during treatment, followed by every 6 months in the 2 years following completion of treatment [13]. There is however a general consensus for discontinuation of TRA and initiation of guideline directed heart failure (HF) therapy which include standard beta-blockers (BB), angiotensin converting enzyme inhibitors (ACE-I) or angiotensin receptor blockers (ARBs) if there is a decrease in LVEF of greater than 15 EF percentage points from baseline [14, 15].

Studies however have demonstrated that cardiotoxicity may be permanent despite discontinuation of TRA and administration of HF therapy [16–18]. As such, there have been initiatives to implement prophylactic use of conventional HF therapy to prevent TIC. There have been both retrospective and prospective reports that have studied use of BB, ACE-I and ARB as cardioprotective agents however the results are still conflicting [19–23].

In this study, we retrospectively obtained data from HER2+ breast cancer patients who were treated with TRA in a rural city in Eastern NC. We hypothesized that use of BB and anti-renin angiotensin aldosterone system (RAAS) agents would be protective against TIC. We also applied a previously published clinical risk score (CRS) [24] to see if it could predict TIC in our real-world patient population.

## Methods and materials

**Fig. 1:**
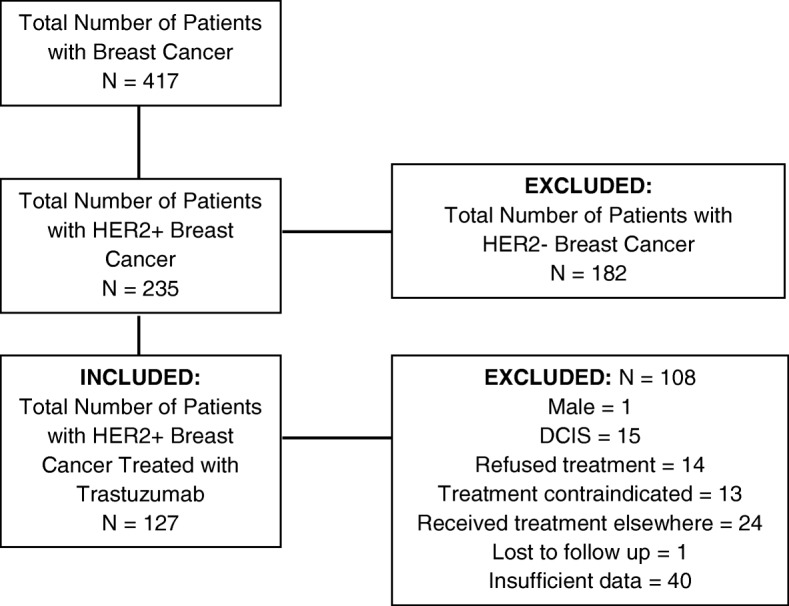
Flow chart of patients included and excluded from the study. A total of 417 patients with HER2+ breast cancer were identified from our institution’s tumor registry. The total number of patients with confirmed HER2+ by pathological and FISH analysis who received TRA was 127. Patients that were excluded totalled 108 as above

Approval of this project titled, ‘Retrospective analysis of cardioprotective effects of cardiac agents in trastuzumab-treated breast cancer patients in Eastern NC was obtained through our Institutional Review Board (UMCIRB 17–000777) at Vidant Medical Center/East Carolina University (VMC/ECU) (Greenville, North Carolina) between 2010 to 2016. HER2+ breast cancer patients were first identified from the Tumor Registry at VMC and associated regional hospitals (Fig. 1). Eligible patients included those who were female, greater than 18 years old, who had received TRA with or without concomitant anthracycline (AC) or non-anthracycline (non-AC) based chemotherapy at our institution or one of our regional hospitals and had a minimum of 2 cardiac imaging studies (echocardiogram or MUGA) at baseline and surveillance (3 months from baseline). Patients were excluded if they were HER2 negative, did not receive TRA, lacked baseline cardiac imaging or did not have a follow up cardiac imaging at 2 to 4 months from baseline, had pre-existing structural heart disease, valvular heart disease, congestive heart failure or cardiomyopathy with an EF < 50%. Data collection which included patient demographics (age, ethnicity and social history), past medical history (presence of hypertension [HTN], diabetes [DM], hyperlipidemia [HLD], dysrhythmias [supraventricular tachycardias and ventricular tachycardias]), AJCC (American Joint Committee on Cancer) breast cancer stage, use and cumulative dosage of AC or non-AC chemotherapy, cardiac medications during TRA treatment for concurrent cardiac history (ex. HTN, HLD, DM) and baseline and surveillance cardiac imaging (echocardiogram and MUGA) was obtained through retrospective chart review. Patients who experienced a decrease of greater than 15 EF percentage points from baseline were defined as those who developed cardiotoxicity (“patients with reduced EF [rEF]”). Patients who did not experience a decrease greater than 15 EF percentage points from baseline were defined as those who did not develop cardiotoxicity (“patients with preserved EF [pEF]”). Demographic data, past medical history, medications and change in EF were compared between the two groups. No adverse or serious adverse events occurred during the study period.

Ezaz and colleagues published a CRS using data from the Surveillance, Epidemiology and End Results (SEER)-Medicare database that stratified patients as low (0–3 points), moderate (4–5 points) and high (≥ 6 points) risk for cardiotoxicity based off on a point system taking into account age, use of AC-based chemotherapy, presence of coronary artery disease (CAD), atrial fibrillation/flutter, DM, HTN and chronic kidney disease (CKD) [24] (Table 1). This CRS was applied to our patient population to test its value as a predictive tool for the detection of cardiotoxicity. Two sensitivity analyses as previously tested in a Canadian setting from patients referred to a cardio-oncology clinic [25] were performed in our patient dataset. The first sensitivity analysis considered a true positive (TP) as a score of ≥4 points (includes both moderate- and high-risk group) and a score of 0–3 points (low-risk group) as a true negative (TN); the second sensitivity analysis considered a TP as a score of ≥6 points (high-risk group only) and a score of 0–5 points (includes both low- and moderate risk) as a TN.

**Table 1:** Risk factors involved in a published clinical risk score for prediction of TIC

Risk Factor	Points
Adjuvant Therapy	
Anthracycline chemotherapy	2
Non-anthracycline chemotherapy	2
No identified chemotherapy	0
Age category, years	
67 to 74	0
75 to 79	1
80 to 94	2
Cardiovascular conditions and risk factors	
Coronary artery disease	2
Atrial fibrillation/flutter	2
Diabetes mellitus	1
Hypertension	1
Renal failure	2

Low risk = 0–3 points, moderate risk = 3–4 points and high risk ≥6 points. Table modified from Ezaz et al., Risk prediction model for heart failure and cardiomyopathy after adjuvant trastuzumab therapy for breast cancer. JAHA 2014:3:e000472

### Statistical analysis

All numerical data are expressed as mean ± standard deviation. Student’s t test was used to determine the significance between continuous variables. Categorical variables are reported as frequencies and proportions. Logistic regression was used to estimate the odds ratio (OR) and the 95% confidence interval (CI) to assess the association of the presence of comorbid risk factors and/or use of cardiac medications with TIC. Age, gender, ethnicity and BMI were included in the multivariable adjusted model. The sensitivity, specificity, positive and negative predictive values of the CRS were calculated using the IBM SPSS Statistics 22.0 statistical analytics software (IBM Corp. Released 2013. Armonk, NY: IBM Corp.). A *p* value of < 0.05 was considered statistically significant.

## Results

### Demographics and breast cancer characteristics of patients treated with TRA

A total of 417 patients with HER2+ breast cancer was identified from our institution’s tumor registry. The total number of patients with confirmed HER2+ by pathological and FISH (fluorescence in-situ hybridization) analysis who received TRA was 127 (Fig. 1).

Of the 127 patients with HER2+ breast cancer who received TRA, 13 (11%) patients developed cardiotoxicity (rEF) while 114 (89%) patients had pEF. The median age was 57 ± 11 years and BMI (body mass index) was 32 ± 8 kg/m^2^. Age, BMI and ethnicity were not significantly different between both groups. In comparison, among patients with rEF, their hormonal status was predominantly ER+/PR+ than ER−/PR- (57% vs 28%, respectively) however this difference was not statistically significant. Patients in both the pEF and rEF groups were predominantly Stage II based on AJCC guidelines. In comparison to the rEF group, a greater proportion of patients with pEF were on a non-AC chemotherapy regimen (docetaxel and cyclophosphamide; paclitaxel and cyclophosphamide) (57% vs 70%, respectively, OR 0.56, 95% CI 0.2–1.7, *p* = 0.32) (Table 2). Patients in the rEF group received more AC based chemotherapy with a cumulative mean dosage of 209.40 ± 54.18 mg/m^2^ (OR 1.76, 95% CI 0.5–5.5).

**Table 2:** Demographic data of HER2+ patients with preserved EF versus decreased EF following TRA exposure

	All Patients	Patients with Preserved EF	Patients with Decreased EF	OR	95% CI	*p* value
Total Number, n (%)	127	114 (89.0)	13 (11.3)	–	–	–
Age (years, mean ± SD)	57 ± 11	57 ± 11	55 ± 10	–	–	0.72
BMI (kg/m^2^, mean ± SD)	32 ± 8	32 ± 12	32 ± 8	–	–	1.0
Ethnicity, n (%)						
Black	64 (50)	57 (50.4)	7 (50)	1.0	0.3–2.8	1.0
White	59 (46)	52 (45.1)	7 (50)	1.19	0.4–3.3	0.75
Hispanic	5 (4)	5 (4.4)	–	–	–	–
Breast Cancer Hormonal Status, n (%)						
ER−/PR-	55 (43)	51 (44)	4 (28)	0.49	0.15–1.66	0.25
ER−/PR+	1 (0.8)	1 (0.8)	–	–	–	–
ER+/PR-	25 (19)	23 (20)	2 (14)	0.66	0.14–3.15	0.60
ER+/PR+	47 (37)	39 (34)	8 (57)	2.56	0.83–7.91	0.10
AJCC Breast Cancer Stage, n (%)						
I	34 (27)	32 (28)	2 (14)	0.43	0.09–2.01	0.28
II	59 (45)	51 (45)	8 (57)	1.64	0.53–5.05	0.38
III	18 (12)	17 (13)	1 (7)	0.44	0.05–3.57	0.44
IV	17 (13)	14 (12)	3 (21)	1.94	0.48–7.85	0.34
Chemotherapy Regimen, n (%)						
AC ➔ TRA	40 (31)	34 (30)	6 (43)	1.76	0.5–5.5	0.32
Non-AC ➔ TRA	88 (68)	80 (70)	8 (57)	0.56	0.2–1.7	0.32
Ejection Fraction (%)						
Baseline (mean ± SD)	64 ± 7	64 ± 7	62 ± 9	–	–	0.16
Median Decrease						
3 months	–	1 ± 7	10 ± 0	–	–	–
6 months	–	3 ± 6	10 ± 10	–	–	–
9 months	–	4 ± 11	17 ± 9	–	–	–
Past Medical History, n (%)						
Arrhythmia	6 (5)	4 (4)	2 (15)	4.58	0.76–27.7	0.09
CAD	8 (6)	6 (5)	2 (15)	3.0	0.54–16.5	0.21
DM	32 (25)	28 (25)	4 (31)	1.22	0.36–4.22	0.74
HTN	78 (61)	69 (61)	9 (69)	1.17	0.37–3.73	0.78
Social History, n (%)						
Smoker	46 (36)	38 (33)	8 (57)	2.67	0.86–8.24	0.08
Alcohol	23 (18)	21 (18)	2 (14)	0.73	0.15–3.55	0.70
Medications, n (%)						
Anti-RAAS	49	47 (32.4)	2 (23)	0.26	0.05–1.22	0.06
BB	24	20 (17.5)	2 (30.7)	0.85	0.17–4.17	0.84
CCB	22	20 (17.5)	2 (15.4)	0.85	0.17–4.17	0.84
Loop diuretic	16	15 (13.1)	1 (7.7)	0.55	0.07–4.50	0.58
Thiazide diuretic	31	29 (25.4)	2 (15.4)	0.48	0.11–2.54	0.37
Statin	25	23 (20.2)	2 (15.4)	0.65	0.15–3.47	0.60

Abbreviations: *AC* anthracycline, *BB* beta-blocker, *CAD* coronary artery disease, *CCB* calcium channel blocker, *CKD* chronic kidney disease, *DM* diabetes mellitus, *ER* estrogen receptor, *HLD* hyperlipidemia, *HTN* hypertension, *OSA* obstructive sleep apnea, *PR* progesterone receptor, *RAAS* renin-angiotensin-aldosterone system. *p* < 0.05 was considered statistically significant

### Development of cardiotoxicity in HER2+ breast cancer patients treated with TRA

**Fig. 2:**
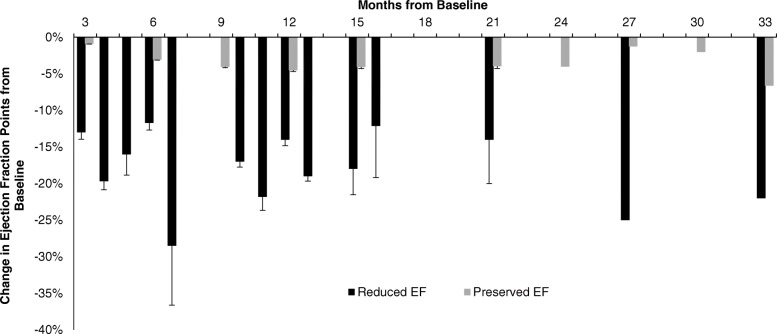
Change in ejection fraction points at surveillance months from baseline (pre-chemotherapy). In patients with who did not experience TIC (pEF), change in EF from baseline at 3-month surveillance intervals was not significantly different. In patients with developed TIC (rEF), a reduction in greater than 15 points from baseline was detected as early as 4 months with overall persistent depression at 15 months from baseline. The reduction in EF was significantly different than pEF at all time points (*p* < 0.05). EF: ejection fraction, pEF: patients who did not develop TIC/preserved EF, rEF: patients who developed TIC/reduced EF

Cardiotoxicity was seen as early as 3 months after initiation of adjuvant TRA therapy and 8 of 13 (62%) patients were symptomatic at the time of diagnosis. Symptoms included palpitations (*n* = 2), dyspnea (*n* = 5) and lower extremity swelling (*n* = 1). Of these 8 symptomatic patients, 2 of the patients were already on BB and ACE-I for concurrent HTN. The remaining 6 patients were referred to Cardiology and started on GDHF therapy. Surveillance EF at 3-month intervals was not significantly different at all time points when compared to baseline in patients who did not experience TIC (pEF). Cardiotoxicity was detected as early as 3 months and a significant decline of greater than 30 EF percentage points from baseline was observed at the 6-month interval. TRA was discontinued in all 14 patients who developed TIC and patients were not re-challenged with TRA. Surveillance interval imaging was increased to 1- to 2-month intervals in patients who developed cardiotoxicity. The EF was found to be persistently depressed from baseline even at the 15-month time point. The reduction in EF was significantly reduced than patients with pEF at all time points (*p* < 0.05) (Fig. 2).

### Co-morbidities and cardiac medications as risk and protective factors in TIC

The presence of co-existing arrhythmia (OR 4.58, 95% CI 0.76–27.7, *p* = 0.09), CAD (OR 3.0, 95% CI 0.54–16.5, *p* = 0.21), HTN (OR 1.17, 95% CI 0.37–3.73, *p* = 0.78) and DM (OR 1.22, 95% CI 0.36–4.22, *p* = 0.74) tended to infer an increased risk for development of cardiotoxicity as did tobacco use (57% vs 33%, OR 2.67, 95% CI 0.86–8.24, *p* = 0.08). Concomitant use of a RAAS inhibitor for a history of HTN during TRA treatment tended to prevent the development of TIC (32% in patients who did not develop reduced EF [pEF] vs 23% in patients who developed TIC [rEF] (OR 0.26, 95% CI 0.05–1.22, *p* = 0.06) (Table 2).

### Predictive value of a clinical risk stratification tool for development of cardiotoxicity

Seventy percent of patients were identified as low risk, 19% as moderate risk and 9% as high risk based on a previously published CRS by Ezaz and colleagues [24] (Table 1). Among patients who were deemed low and moderate risk, 4% developed cardiotoxicity in comparison to 1% that were deemed high risk (Table 3). A sub-analysis of the 6 patients from the low-risk group revealed that 0% of these patients were on a RAAS inhibitor or BB (metoprolol and carvedilol). In comparison, among the 10 patients from the high-risk group who did not develop cardiotoxicity 42 and 40% of these patients were on a RAAS inhibitor and BB concurrently for a history of HTN, respectively (Table 3 b). For patients with a CRS of ≥4 points (moderate- and high-risk), the sensitivity was 0.19 (95% CI 0.09–0.36), specificity was 0.92 (95% CI 0.84–0.97), positive predictive value (PPV) was 0.50 (95% CI 0.25–0.76) and negative predictive value (NPV) 0.74 (95% CI 0.64–0.81). For patients with a CRS of ≥6 points (high-risk), the sensitivity was 0.17 (95% CI 0.03–0.49), specificity was 0.89 (95% CI 0.82–0.94), PPV was 0.14 (95% CI 0.03–0.44) and NPV 0.91 (95% CI 0.84–0.95) (Table 4).

**Table 3:** Cardiotoxicity events by clinical risk score

	Clinical Risk Score		
	Low (0–3)	Moderate (4–5)	High (≥ 6)
Patients (n)	90 (70)	25 (19)	12 (9)
Cardiotoxicity [n (%)]	6 (4)	5 (4)	2 (1)
Permanent reduced EF [n (%)]	5 (4)	2 (1)	1 (0.1)

**Table 4:** Sensitivity analyses of the clinical risk score

	Clinical Risk Score			
	Moderate + High Risk (≥ 4)		High Risk (≥ 6)	
	Value	95% CI	Value	95% CI
Sensitivity	0.19	0.09–0.36	0.17	0.03–0.49
Specificity	0.92	0.84–0.97	0.89	0.82–0.94
Positive predictive value	0.50	0.24–0.76	0.14	0.03–0.44
Negative predictive value	0.74	0.64–0.81	0.91	0.84–0.95

## Discussion

In our retrospective study of a real world heterogenous population of HER2+ breast cancer patients receiving TRA, we demonstrate that concomitant use of RAAS inhibitors tended to infer a decreased risk of TIC. This was further supported by a sub-analysis of patients who developed TIC despite being categorized as low-risk based off a previously published CRS [24].

Among the different concomitant cardiac medications, patients who did not experience TIC were more commonly on a RAAS inhibitor for a history of HTN. There was a higher use of BB in patients who did not experience TIC however this never achieved statistical significance. Previous studies have shown promising effects with carvedilol which may be due to its partial anti-inflammatory effects [26, 27] in comparison to metoprolol. The majority of our patients were on metoprolol (data not shown) and this may explain the non-significant effect of BB as cardioprotection in our patient population. Published data from a retrospective study concluded a unique time-dependent effect of combined BB, ACE-I/ARB on LVEF in patients receiving TRA for breast cancer [28]. In patients receiving concomitant BB, ACE-I/ARB with TRA, a decline in EF was initially observed at 3 months, however EF improved between 3 and 12 months in comparison to patients receiving TRA alone [28].

Since then, there have been prospective studies that tested the use of an ACE-I, ARB and BB as cardioprotective against TIC however with conflicting results. In the MANTICORE 101-Breast Study (Multidisciplinary Approach to Novel Therapies in Cardio-Oncology Research [19], perindopril and bisoprolol were found to protect against TRA-induced decrease in EF by a change in − 3% and − 1%, respectively, vs placebo of − 5%. Their primary endpoint of TRA-mediated LV remodeling based off of LV end-diastolic volume indices however was not significantly affected by either of these drugs. It is important to note however that a significant proportion of their patient population were younger, had fewer CVD risk factors and did not receive AC chemotherapy. Therefore, the protective effects of the studied therapies may have been masked by left ventricles that did not experience the ‘dual hit’ of TRA and AC. In a small randomized control trial in the Netherlands, the effect of candesartan at its maximum dose in TIC was shown to be ineffective for protection against TIC [20]. It has been argued that the study design was flawed because of its unconventional dosing strategy, younger and healthier population that were not exposed to prior AC-therapy. In another intention-to-treat study by Gulati and colleagues, candesartan was found to provide protection from early decline in LVEF in comparison to metoprolol irrespective of TRA use [21]. Recent results from a phase II prospective 2-year clinical trial in HER2+ breast cancer patients by Guglin and colleagues revealed that lisinopril and carvedilol appeared to offer a protective effect by attenuation of decline in EF in patients receiving combination AC and TRA [22, 23]. This benefit was not observed in patients receiving TRA alone and as such supports the “dual hit” combined TRA and AC cardiotoxicity hypothesis. Indeed, our results were suggestive of this as our patients who received AC-based chemotherapy tended to develop cardiotoxicity more frequently than patients who received non-AC based regimen. Other identified risk factors for TIC include older age, HTN, DM and smoking [8, 29] however this has also been disputed elsewhere [30, 31].

In vitro experiments have demonstrated that severe cardiac dysfunction occurs following AC therapy with adjunctive TRA secondary to impaired recovery mechanisms of HER signaling in the setting of AC-induced reactive oxygen species (ROS) cardiotoxicity [32, 33]. One such mechanism is via an increase in angiotensin II (AngII) in response to AC-induced stress. The effects of AngII on the myocardium are two-fold: direct ROS production through the NADPH oxidase system [34, 35] as well as serving as an inhibitor of neuregulin 1 (NRG1), an essential ligand involved with HER2 survival signaling [32]. AngII activation of these detrimental downstream cascades can be promoted with beta-adrenergic stimulation. In fact, in a small study of 15 HER2+ breast cancer patients, serum levels of norepinephrine and blood pressure were found to be elevated with an associated decrease in EF within three months of TRA treatment [36]. The true protective effect of agents targeting the RAAS are thus still in heavy debate with additional clinical trials in much need.

TIC occurred in 11% of our patients which is similar to reports of cardiac dysfunction in 7 to 18% of patients [6–8] but was considerably less than previously reported retrospective studies [30, 31, 28]. This may be due to differences in population and definition of cardiotoxicity. Although it has been suggested that TIC may be reversible with conventional HF management or with TRA cessation, several other studies have demonstrated otherwise [16–18]. Up to 22% of patients required immediate removal of TRA due to progressive LV dysfunction despite appropriate cardiac pharmacotherapy with some experiencing long-term adverse effects from the drug [17, 37]. In our patients who developed TIC, a persistent decrease in EF greater than 10 percentage points from baseline at 15-month follow up was observed despite initiation of GDHF therapy.

A CRS for early prediction of cardiotoxicity has been explored as this may help with determining aggressiveness of chemotherapy and identifying patients who require more intensive versus lenient cardiac monitoring. Currently there are two CRS that have been published: the first in the seven-year follow-up of the NSABP-31 clinical trial [38] and a more recent score by Ezaz and colleagues [24]. In comparison to a study by Rushton et al who tested the use of the CRS in a Canadian patient population [25], there was a substantially smaller percentage of patients in our study who developed cardiotoxicity (43% vs 11%, respectively). This discrepancy may have been due to the differences in definition of cardiotoxicity in addition to the fact that these patients were already high risk as they were referred to a cardio-oncology clinic. The CRS was derived from the SEER database of which patients were much older (73.6 +/− 5.3 years) and predominantly Caucasian [24]. In comparison, our patients were younger (57 years) and were equally African American and Caucasian. The low sensitivity and positive predictive value of the CRS may miss patients at high risk of developing TIC and consequently more intensive surveillance is recommended. The discrepant observation in risk stratification and actual cardiotoxic events may be explained by the presence of RAAS inhibitors. Among the 10 patients that did not develop cardiotoxicity who were deemed high risk, 47% of those patients were concurrently taking a RAAS inhibitor for their history of HTN. Further supporting this observation, all 6 patients who were identified as low-risk were not on a RAAS inhibitor. In addition, the CRS was designed to be used prospectively and therefore may not have good sensitivity when used retrospectively as in our study.

### Limitations

Limitations of this study include firstly that this was a retrospective design and a small sample size from a single institution. Our definition of cardiotoxicity was based on FDA guidelines of TRA and this may have resulted in a lower incidence of TIC. Recent literature has demonstrated the utility of global longitudinal strain (GLS) in addition to three-dimensional echocardiography with speckle tracking imaging (STI) as an early predictor of LV dysfunction in patients receiving TRA that may serve as a better tool for identifying patients at risk for TIC [39]. These novel echocardiographic parameters may have also been useful to assess right ventricular (RV) function as there has been some data demonstrating deleterious effects of AC and TRA on RV systolic and diastolic function [40]. As this was a retrospective study, these unique echocardiographic parameters for detection of monitoring of TIC were not uniformly applied to each individual patient. Post-processing software for GLS was not available at our institution and incidence of TIC may have been different if GLS and STI were used rather than solely on a decrease in EF percentage points. The chemotherapy regimen was also not standardized; a small proportion of patients who also received combination TRA and pertuzumab/lapatinib which has less cardiotoxic effects [41] which may have contributed to lower TIC incidence. Importantly, while we acknowledge that it is difficult to determine if cardiotoxicity observed in our patient population was also due to AC, mean cumulative dosage of AC was on the lower threshold end of cardiotoxicity (209.40 ± 54.18 mg/m^2^ vs 450–500 mg/m^2^) [42, 43]. This further supports the ‘dual hit’ hypothesis of AC and TRA administration in the development of TIC. Medications of the ACE-I and ARB were grouped as RAAS inhibitors and therefore individual effects of either class could not be identified.

## Conclusion

In summary, TIC is an ongoing problem for the treatment of HER2+ breast cancer with uncertain long-term outcomes that may result in associated detrimental consequences of HF. Medications inhibiting RAAS may be cardioprotective likely via survival signaling via the NRG-1/HER pathways. The CRS may be applicable in a real-world population to identify high risk patients that require more aggressive monitoring. Future large clinical trials testing specific anti-RAAS agents for protection of TIC and the involved cellular mechanisms are further warranted.

## Data Availability

All the relevant data and material are presented in the main manuscript.

## Abbreviations

AC: Anthracycline
ACE-I: Angiotensin converting enzyme inhibitor
AJCC: American Joint Committee on Cancer
AngII: Angiotensin II
ARB: Angiotensin receptor blocker
BB: Beta blocker
BMI: Body mass index
CAD: Coronary artery disease
CI: Confidence interval
CKD: Chronic kidney disease
CRS: Clinical risk score
CVD: Cardiovascular disease
DM: Diabetes mellitus
EF: Ejection fraction
ER: Estrogen receptor
FDA: Food and drug administration
FISH: Fluorescence in-situ hybridization
GDHF: Goal directed heart failure
GLS: Global longitudinal strain
HER2: Human epidermal growth factor receptor 2
HF: Heart failure
HLD: Hyperlipidemia
HTN: Hypertension
LV: Left ventricle
LVEF: Left ventricular ejection fraction
MANTICORE: Multidisciplinary Approach to Novel Therapies in Cardio-Oncology Research
MUGA: Multigated acquisition scan
NADPH: Reduced nicotinamide adenine dinucleotide phosphate
NPV: Negative predictive value
NRG1: Neuregulin 1
OR: Odds ratio
pEF: Patients with preserved EF
PPV: Positive predictive value
PR: Progesterone receptor
RAAS: Renin angiotensin aldosterone system
rEF: Patients with reduced EF
ROS: Reactive oxygen species
RV: Right ventricle
SEER: Surveillance, Epidemiology and End Results
STI: Speckle tracking imaging
TIC: Trastuzumab-induced cardiotoxicity
TN: True negative
TP: True positive
TRA: Trastuzumab
VMC/ECU: Vidant Medical Center/East Carolina University

## References

[1] Schechter AL et al. The neu oncogene: an erb-B-related gene encoding a 185,000-Mr tumour antigen. 1984; 312:513–516. 10.1038/312513a0 6095109

[2] Owens MA (2004). HER2 amplification ratios by fluorescence in situ hybridization and correlation with immunohistochemistry in a cohort of 6556 breast cancer tissues. Clin Breast Cancer.

[3] Moasser MM (2007). The oncogene HER2: its signaling and transforming functions and its role in human cancer pathogenesis. Oncogene.

[4] Garnack-Jones KP (2010). Trastuzumab: a review of its use as adjuvant treatment in human epidermal growth factor receptor 2 (HER-2)-positive early breast cancer. Drugs.

[5] Slamon D (2001). Use of chemotherapy plus a monoclonal antibody against HER2 for metastatic breast cancer that overexpresses HER2. NEJM.

[6] Piccart-Gebhart MJ (2005). Trastuzumab after adjuvant chemotherapy in HER2-positive breast cancer. NEJM.

[7] Perez (2014). Trastuzumab plus adjuvant chemotherapy for human epidermal growth factor receptor 2-positive breast cancer: planned joint analysis of overall survival from NSABP B-31 and NCCTG N9831. J Clin Oncol.

[8] Slamon (2011). Adjuvant trastuzumab in HER2-positive breast cancer. NEJM.

[9] Bowles EJ (2012). Risk of heart failure in breast cancer patients after anthracycline and trastuzumab treatment: a retrospective cohort study. J Natl Cancer Inst.

[10] Zeglinski K (2011). Trastuzumab-induced cardiac dysfunction: a ‘dual hit. Exp Clin Cardiol.

[11] Askoxylakis V (2010). Long-term survival of cancer patients compared to heart failure and stroke: a systematic review. BMC Cancer.

[12] Nowsheen S (2018). Trastuzumab in female breast cancer patients with reduced LVEF. J Am Heart Assoc.

[13] Armenian SH (2017). Prevention and monitoring of cardiac dysfunction in survivors of adult cancers: American society of clinical oncology clinical practice guideline. J Clin Oncol.

[14] Mackey JR (2008). Cardiac management during adjuvant trastuzumab therapy: recommendations of the Canadian Trastuzumab working group. Curr Oncol.

[15] Jones AL (2009). Management of cardiac health in trastuzumab-treated patients with breast cancer: updated United Kingdom National Cancer Research Institute recommendations for monitoring. Brit J Cancer.

[16] Cardinale D (2010). Trastuzumab-induced cardiotoxicity: clinical and prognostic implications of troponin I elevation. J Clin Oncol.

[17] Telli ML (2007). Trastuzumab-related cardiotoxicity: calling into question the concept of reversibility. J Clin Oncol.

[18] Wadhwa D (2009). Trastuzumab mediated cardiotoxicity in the setting of adjuvant chemotherapy for breast cancer: a retrospective study. Breast Cancer Res Treat.

[19] Pituskin E (2017). Multidisciplinary approach to novel therapies in cardio-oncology research (MANTICORE 101-breast): a randomized trial for the prevention of trastuzumab-associated cardiotoxicity. J Clin Oncol.

[20] Boekhout A (2016). Angiotensin II-receptor inhibition with candesartan to prevent trastuzumab-related cardiotoxic effects in patients with early breast cancer. JAMA Oncol.

[21] Gulati G (2016). Prevention of cardiac dysfunction during adjuvant breast cancer therapy (PRADA): a 2x2 factorial, randomized, placebo-controlled, double-blind clinical trial of candesartan and metoprolol. Eur Heart J.

[22] Guglin M (2019). Randomized trial of lisinopril versus carvedilol to prevent trastuzumab cardiotoxicity in patients with breast cancer. JACC.

[23] Guglin M (2017). Lisinopril or coreg CR in reducing cardiotoxicity with breast cancer receiving trastuzumab: a rationale and design of a randomized clinical trial. Am Heart J.

[24] Ezaz G (2014). Risk prediction model for heart failure and cardiomyopathy after adjuvant trastuzumab therapy for breast cancer. J Am Heart Assoc.

[25] Rushton M (2017). Trastuzumab-induced cardiotoxicity: testing a clinical risk score in a real-world cardio-oncology population. Curr Oncol.

[26] Wang D (2014). Carvedilol has stronger anti-inflammation and anti-virus effects than metoprolol in murine model with coxsackie virus B3-inducd viral myocarditis. Gene.

[27] Avila Mônica Samuel, Ayub-Ferreira Silvia Moreira, de Barros Wanderley Mauro Rogerio, das Dores Cruz Fatima, Gonçalves Brandão Sara Michelly, Rigaud Vagner Oliveira Carvalho, Higuchi-dos-Santos Marília Harumi, Hajjar Ludhmila Abrahão, Kalil Filho Roberto, Hoff Paulo Marcelo, Sahade Marina, Ferrari Marcela S.M., de Paula Costa Romulo Leopoldo, Mano Max Senna, Bittencourt Viana Cruz Cecilia Beatriz, Abduch Maria Cristina, Lofrano Alves Marco Stephan, Guimaraes Guilherme Veiga, Issa Victor Sarli, Bittencourt Marcio Sommer, Bocchi Edimar Alcides (2018). Carvedilol for Prevention of Chemotherapy-Related Cardiotoxicity. Journal of the American College of Cardiology.

[28] Oliva S (2012). Administration of angiotensin-converting enzyme inhibitors and β-blockers during adjuvant trastuzumab chemotherapy for nonmetastatic breast cancer: marker of risk of cardioprotection in the real world?. Oncologist.

[29] Procter M (2010). Longer-term assessment of trastuzumab-related cardiac adverse events in the herceptin adjuvant (HERA) trial. J Clin Oncol.

[30] Farolfi A (2013). Trastuzumab-induced cardiotoxicity in early breast cancer patients: a retrospective study of possible risk and protective factors. Heart.

[31] Naumann D (2013). Factors predicting trastuzumab-related cardiotoxicity in a real-world population of women with HER2+ breast cancer. Anticancer Res.

[32] Sawyer DB (2002). Modulation of anthracycline-induced myofibrillar disarray in rat ventricular myocytes by neuregulin-1β and anti-erbB2. Circulation.

[33] Timolati F (2006). Neuregulin-1 beta attenuates doxorubicin-induced alterations of excitation coupling and reduces oxidative stress in adult rat cardiomyocytes. J Mol Cell Cardiol.

[34] Nakagami H (2003). NADPH oxidase-derived superoxide anion mediates angiotensin II-induced cardiac hypertrophy. J Mol Cell Cardiol.

[35] Mollanu H (2005). Mechanisms of increased vascular superoxide production in an experimental model of idiopathic dilate cardiomyopathy. Arterioscler Thromb Vasc Biol.

[36] Lenneman CG (2014). Sympathetic nervous system alterations with HER2+ antagonism: an early marker of cardiac dysfunction with breast cancer treatment?. Ecancermedicalscience.

[37] Ewer MS (2007). Cardiac toxicity of trastuzumab-related regimens in HER2-overexpressing breast cancer. Clin Breast Cancer.

[38] Romond EH (2012). Seven-year follow-up assessment of cardiac function in NSABP B-31, a randomized control trial comparing doxorubicin and cyclophosphamide followed by paclitaxel (acp) with acp plus trastuzumab as adjuvant therapy for patients with node-positive, human epidermal growth factor receptor 2–positive breast cancer. J Clin Oncol.

[39] Gripp EA (2018). Global longitudinal strain accuracy for cardiotoxicity prediction in a cohort of breast cancer patients during anthracycline and/or trastuzumab treatment. Arq Bras Cardiol.

[40] Tadic (2017). The influence of chemotherapy on the right ventricle: did we forget something?. Clin Cardiol.

[41] Sendur MA (2013). Cardiotoxicity of novel HER2-targeted therapies. Curr Med Res Opin.

[42] Van Hoff DD (1979). Risk factors for doxorubicin-induced congestive heart failure. Ann Intern Med.

[43] Rahman AM (2007). Anthracycline-induced cardiotoxicity and the cardiac-sparing effect of liposomal formulation. Int J Nanomedicine.

